# KEY INSTRUMENTAL ACTIVITIES OF DAILY LIVING AND COGNITIVE DOMAINS IN NON-DEMENTED OLDER ADULTS: A NETWORK ANALYSIS

**DOI:** 10.1093/geroni/igae098.4011

**Published:** 2024-12-31

**Authors:** Jiaying Li, Rendong He, Erh-Chi Hsu, Junxin Li

**Affiliations:** Johns Hopkins University, Baltimore, Maryland, United States; Jilin University School of Nursing, Changchun, Jilin, China; Johns Hopkins University, Baltimore, Maryland, United States; Johns Hopkins School of Nursing, Baltimore, Maryland, United States

## Abstract

**Background:**

Interconnected relationships within and between instrumental activities of daily living (IADL) and cognitive domains indicate central IADLs, cognitive domains, and bridge IADLs, which we aim to identify as modifying them can effectively enhance holistic cognitive and IADL functions.

**Methods:**

A cross-sectional analysis of adults aged 65 and older in United States on five IADL and six cognitive domains. Network analysis identified central and bridge variables. Non-parametric and case-dropping bootstrap methods checked network stability. Network comparison tests assessed sex differences with Benjamini-Hochberg adjustments.

**Results:**

Of the 2,239 participants, 56.4% were female. We computed and tested three networks: IADL, cognition, and bridge. The Correlation Stability Coefficients for these were 0.67, 0.75, and 0.44 respectively, all exceeding the threshold of 0.25. Meal preparation (centrality: 3.87), visual attention (0.86), and shopping (0.41) were identified as central IADL, central cognition, and bridge IADL respectively, as they had the highest centrality indices and were significantly higher than others in their networks (p < 0.05). Notably, gender differences emerged in the IADL network, with stronger associations between laundry and meal preparation in females (1.69 vs. males: 0.74, p = 0.001) and higher centrality in meal preparation among females (difference = 1.99, p = 0.007).

**Conclusion:**

While broad enhancements in all IADL and cognitive domains are beneficial, targeting meal preparation, visual attention, and shopping optimizes cost-effectiveness in improving holistic IADL, holistic cognition, and holistic cognition function via IADL interventions among older adults. Notably, meal preparation interventions may be less effective in males, requiring tailored approaches.

